# Addisonian Crisis Mimicking Acute Kidney Injury in Dogs: A Retrospective Study of 34 Dogs Diagnosed with Acute Kidney Injury in Romania

**DOI:** 10.3390/life16010127

**Published:** 2026-01-14

**Authors:** Ștefania Roșca, Gheorghe Solcan, Mihail Moroz, Raluca Adriana Ștefănescu, Alina Levința, Paula Maria Pașca

**Affiliations:** 1 Faculty of Veterinary Medicine, “Ion Ionescu de la Brad” Iasi University of Life Sciences, M. Sadoveanu Alley 8, 700489 Iasi, Romania; stefania.rosca@iuls.ro (Ș.R.); raluca.stef@yahoo.ro (R.A.Ș.); alina.levinta@iuls.ro (A.L.); paula.pasca@iuls.ro (P.M.P.); 2 Faculty of Veterinary Medicine, Technical University of Moldova, MD-2004 Chisinau, Moldova; mihail.moroz@sfc.utm.md

**Keywords:** hypoadrenocorticism, dog, acute kidney injury, diagnostic, treatment

## Abstract

Primary hypoadrenocorticism (Addison’s disease) is an uncommon but potentially life-threatening endocrine disorder in dogs. Affected animals may present with clinicopathological features mimicking acute kidney injury (AKI). The challenge in diagnosing hypoadrenocorticism arises from its highly heterogeneous and non-specific clinical presentation, including acute kidney injury (AKI). This retrospective observational study aimed to evaluate dogs presenting with AKI and to identify cases in which primary hypoadrenocorticism was the underlying etiology. Thirty-four dogs diagnosed with acute kidney injury were evaluated at the Clinical Hospital for Companion Animals of the “Ion Ionescu de la Brad” University of Life Sciences, Iași, Romania, among which three (8.8%) were endocrinologically confirmed to have primary hypoadrenocorticism. The evaluation protocol included a complete clinical examination, hematological, biochemical, and hormonal investigations, urinalysis, abdominal ultrasonography, and an ACTH stimulation test. These dogs exhibited hyponatremia, hyperkalemia, a reduced sodium-to-potassium ratio, and azotemia at admission, closely resembling intrinsic AKI. Following fluid therapy and hormone replacement, rapid normalization of electrolyte and renal parameters was observed. These findings support hypovolemia and electrolyte imbalance as the primary mechanisms underlying reversible prerenal azotemia in these cases. If not diagnosed early, this condition has a significant risk of progressing to acute tubular necrosis. The findings highlight the need for careful differentiation between primary AKI and renal dysfunction secondary to Addison’s disease, as well as the importance of promptly initiating hormone replacement therapy. In conclusion, hypoadrenocorticism should be considered in dogs presenting with AKI and electrolyte imbalance. Early endocrine evaluation and prompt initiation of targeted therapy are essential to avoiding misdiagnosis and optimizing clinical outcomes.

## 1. Introduction

Canine hypoadrenocorticism (Addison’s disease) is a relatively uncommon endocrinopathy, with an estimated prevalence of less than 0.1% in the canine population, yet it carries major clinical significance due to its potentially life-threatening nature and the considerable challenges associated with differential diagnosis [1,2]. The disorder is characterized by functional insufficiency of the adrenal cortex, leading to a deficiency of glucocorticoids (primarily cortisol) and mineralocorticoids (mainly aldosterone)—hormones that are essential for maintaining electrolyte balance, blood volume homeostasis, and the metabolic response to stress [3,4,5,6].

Aldosterone secretion is primarily regulated by circulating potassium and angiotensin II levels, while ACTH plays only a minor stimulatory role. Conversely, cortisol production is tightly controlled by corticotropin-releasing hormone (CRH) and ACTH concentrations. Cortisol is essential for glucose homeostasis through gluconeogenesis, glycogen synthesis, lipolysis, and protein catabolism, and it modulates calcium–phosphate metabolism, water balance, gastrointestinal function, cardiovascular performance, and hematopoiesis while suppressing immune and inflammatory responses [7]. Aldosterone maintains water, acid–base, and electrolyte homeostasis by promoting sodium reabsorption and potassium and hydrogen ion excretion, predominantly in the distal nephron. Primary hypoadrenocorticism results from adrenocortical destruction leading to glucocorticoid and mineralocorticoid deficiency, most often immune-mediated, though neoplasia, infections, infarction, or drug-induced causes (e.g., trilostane, mitotane) have been described [6,8,9]. Secondary and tertiary (central) forms arise from impaired ACTH or CRH secretion due to pituitary or hypothalamic pathology, trauma, neoplasia, or iatrogenic suppression following glucocorticoid therapy [10]. Most dogs with primary disease exhibit deficiencies of both hormone types and corresponding electrolyte abnormalities, although isolated cortisol deficiency with preserved mineralocorticoid function can occur due to selective cortical destruction or compensatory mechanisms maintaining electrolyte balance [11,12]. Some cases initially present with normal electrolytes owing to symptomatic correction or early disease and later progress to classical Addisonian profiles [13,14]. Mineralocorticoid deficiency without glucocorticoid insufficiency has rarely been reported, although incomplete evaluation or hyporeninemic hypoaldosteronism could not always be excluded [15,16].

The clinical presentation of canine hypoadrenocorticism is highly variable and often non-specific, which explains its frequent designation in veterinary medicine as “the great pretender” [1,17,18]. The most commonly reported clinical signs include lethargy, anorexia, vomiting, diarrhea, bradycardia, atrioventricular block, dehydration, and episodic collapse—manifestations that can easily be mistaken for other acute syndromes, particularly acute kidney injury (AKI) [9,19].

A key diagnostic challenge arises from the striking biochemical similarity between Addisonian crisis and acute kidney injury. Common laboratory abnormalities include azotemia, hyperkalemia, hyponatremia, hyperphosphatemia, and a decreased glomerular filtration rate [5,20,21]. In most cases, these alterations reflect a functional prerenal insufficiency with a favorable prognosis if early diagnosis and appropriate therapy are promptly instituted. However, delayed recognition can lead to renal ischemia and progression to intrinsic acute kidney injury associated with acute tubular necrosis, resulting in a guarded outcome [21,22].

Acute kidney injury (AKI) represents a heterogeneous clinical syndrome that may arise from prerenal, renal, or postrenal mechanisms [23,24]. In dogs with hypoadrenocorticism, hypovolemia and aldosterone deficiency result in reduced renal perfusion and functional prerenal azotemia, which can mimic intrinsic renal disease in the absence of structural nephropathy [5,6,7,19]. Consequently, reliance on azotemia severity alone may lead to misclassification of Addisonian crisis as primary AKI.

Electrolyte disturbances, particularly hyponatremia, hyperkalemia, and a decreased sodium-to-potassium (Na/K) ratio, are commonly associated with primary hypoadrenocorticism and are frequently used as screening indicators [7,13,17]. However, a reduced Na/K ratio lacks disease specificity and has been reported in several non-endocrine critical conditions, including severe hypovolemia, systemic illness, and postrenal disorders [14,17]. Interpretation of electrolyte patterns without appropriate clinical context and confirmatory endocrine testing may therefore delay accurate diagnosis and appropriate therapy.

Delayed recognition of Addisonian crisis may have important clinical consequences. Inadequate or postponed hormone replacement therapy can prolong hypovolemia and electrolyte imbalance, increasing the risk of progression from reversible prerenal azotemia to intrinsic acute kidney injury with tubular damage [5,21]. In contrast, early diagnosis and targeted treatment are associated with rapid correction of biochemical abnormalities and favorable outcomes [18,22].

Despite its clinical relevance, comparative data evaluating dogs with Addisonian crisis-associated AKI alongside dogs with non-endocrine AKI remain limited. Most published reports focus on isolated case descriptions or small series, while systematic comparative analyses of clinical, biochemical, and diagnostic features are scarce.

The aim of this study was therefore to retrospectively evaluate dogs presenting with suspected acute kidney injury and to compare clinical, biochemical, hematological, and imaging findings between dogs with Addisonian crisis-associated AKI and those with non-endocrine AKI to improve diagnostic accuracy and clinical decision-making.

## 2. Materials and Methods

The investigation was performed between January and September 2025 at the Clinical Hospital for Companion Animals, “Ion Ionescu de la Brad” University of Life Sciences, Iași, Romania. This investigation was designed as a retrospective observational cohort study including 34 dogs evaluated for suspected acute kidney injury (AKI), with cases subsequently classified as Addisonian crisis-associated AKI or non-endocrine AKI based on endocrine testing and final clinical diagnosis. Among these, three cases (8.8%) were endocrinologically confirmed as primary hypoadrenocorticism (Addison’s disease), based on clinical presentation, biochemical findings, and endocrine testing criteria. These cases represented a rare but clinically significant etiology of AKI. The remaining thirty-one dogs were diagnosed with AKI and were investigated to identify concurrent comorbidities, such as babesiosis, acute pancreatitis, systemic inflammatory response syndrome, and ethylene glycol intoxication.

### 2.1. Inclusion and Exclusion Criteria

The selection of patients was based on the following inclusion criteria: the presence of a clinical picture suggestive of AKI or hypoadrenocorticism (lethargy, inappetence, vomiting, diarrhea, bradycardia, dehydration, or collapse), correlated with characteristic biochemical abnormalities such as hyponatremia, hyperkalemia, azotemia, and hyperphosphatemia. The endocrinological diagnosis was confirmed through the adrenocorticotropic hormone (ACTH) stimulation test. Exclusion criteria included the presence of acute or chronic systemic diseases that could influence renal function or hormonal parameters.

### 2.2. Diagnostic and Evaluation Protocol

All patients included in the study underwent a comprehensive clinical examination and a standardized paraclinical diagnostic protocol, which encompassed hematological, biochemical, urinary, imaging, and hormonal investigations. A summary of the diagnostic methods employed is presented in Table 1.

### 2.3. Sample Collection and Laboratory Analysis

At the time of presentation at the clinic, biological samples (blood and urine) were collected for comprehensive clinical and paraclinical evaluation. The sampling procedure was performed in accordance with standard protocols of good veterinary practice, adhering to aseptic and biosafety guidelines. The handling, preservation, and transport of samples were carried out under controlled conditions to maintain the integrity of physiological parameters and ensure the accuracy of subsequent analyses.

*Biochemical Parameters:* To assess metabolic status and organ function, blood samples were collected via venipuncture into plain vacuum tubes (red-top, with clot activator). Following centrifugation, serum samples were analyzed using automated analyzers (Abaxis VetScan VS2, (Zoetis Services, Parsippany, NJ, USA); BioSystems BA200, Barcelona, Spain, and Diestro Electrolyte Analysers 103 AP V4 Semi Basic, Buenos Aires, Argentina). The biochemical profile included the evaluation of key metabolic, renal, and electrolyte parameters relevant to the diagnosis of AKI or primary hypoadrenocorticism.

*Hematological Parameters:* To assess hematological status, whole blood samples were analyzed using the automated hematology analyzer Abaxis VetScan HM5 (Abaxis, Union City, CA, USA). The measured parameters included total erythrocyte, leukocyte, and platelet counts; hemoglobin (HGB); hematocrit (HCT); mean corpuscular volume (MCV); mean corpuscular hemoglobin (MCH); and mean corpuscular hemoglobin concentration (MCHC).

*Endocrine Testing:* To confirm the diagnosis of primary hypoadrenocorticism, a synthetic ACTH (Cosacthen^®^) stimulation test was performed, which is considered the gold standard for evaluating adrenocortical function in dogs. The protocol involved the collection of a baseline blood sample, followed by intravenous administration of Cosacthen^®^ at a dose of 5 µg/kg. A second blood sample was obtained 60 min post-administration for measurement of serum cortisol concentration. Cortisol levels were determined using a Vcheck^®^ immunoassay analyzer (Bionote, Hwaseong, Republic of Korea).

*Urinary Biochemistry:* To assess renal function and electrolyte balance, urine samples were analyzed using the automated analyzer Abaxis VetScan UA14 (Zoetis Services, Parsippany, NJ, USA). The urinalysis profile included measurement of urine specific gravity, pH, proteinuria, glucosuria, ketonuria, and the presence of bilirubin and blood. These results were complemented by a microscopic examination of the urinary sediment to identify cellular elements, crystals, and casts.

### 2.4. Imaging Investigations

Abdominal ultrasonographic examination was performed using a GE Logiq V5 system (GE Healthcare, Chicago, IL, USA) equipped with a multifrequency 5–10 MHz transducer suitable for evaluating parenchymal structures in dogs. The scanning protocol followed the principles of the AFAST (Abdominal Focused Assessment with Sonography for Trauma) method and was expanded to facilitate detailed assessment of the adrenal glands, kidneys, and other abdominal organs.

### 2.5. Data Analysis and Clinical Monitoring

The clinical and paraclinical progression of patients diagnosed with primary hypoadrenocorticism was monitored throughout hospitalization and during the immediate post-therapeutic period. Hematological and biochemical parameters were periodically re-evaluated to assess clinical progress and response to the instituted treatment.

The longitudinal assessment included dynamic monitoring of electrolyte balance (Na^+^, K^+^, Cl^−^), renal function (urea, creatinine), and hematological profile, with particular attention to the correction of anemia and restoration of blood volume.

All collected data were comparatively analyzed over time to evaluate the effectiveness of hormone replacement therapy and supportive treatment in restoring metabolic and circulatory homeostasis.

## 3. Results

### 3.1. Biochemical and Hematological Evolution

**Figure 1:**
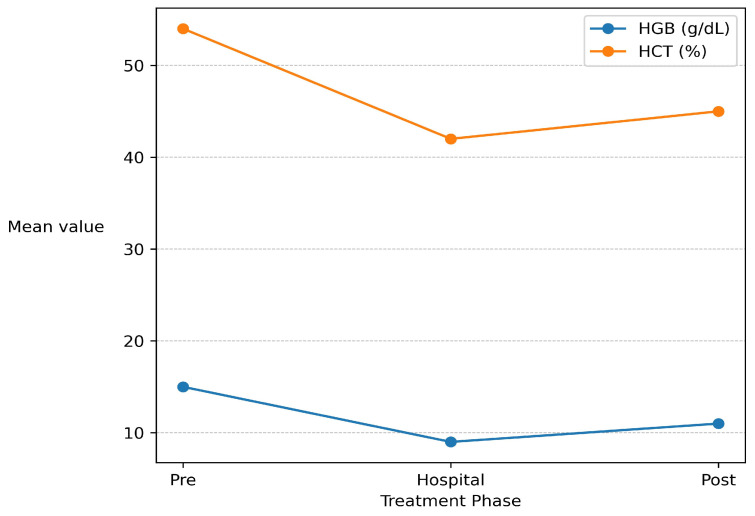
Evolution of hematological parameters across treatment phases.

At baseline (pre-phase), all three canine cases presented with a constellation of biochemical and hematological alterations consistent with Addisonian crisis complicated by prerenal azotemia. The biochemical profile was characterized by marked hyponatremia (127.8 ± 3.12 mmol/L) and hyperkalemia (6.72 ± 0.99 mmol/L), resulting in a decreased Na^+^/K^+^ ratio (19.68 ± 1.74)—a hallmark indicator of primary adrenocortical insufficiency. Simultaneous increases in blood urea nitrogen (130.37 ± 109.82 mg/dL), creatinine (3.05 ± 2.25 mg/dL), and phosphorus (9.67 ± 3.11 mg/dL) reflected prerenal azotemia secondary to hypovolemia and impaired renal perfusion. Hematological evaluation (Figure 1) demonstrated elevated hemoglobin (18.60 ± 3.67 g/dL) and hematocrit (54.04 ± 12.24%), indicating hemoconcentration prior to fluid resuscitation.

During hospitalization (hospital treatment phase), electrolyte disturbances were progressively corrected under combined fluid therapy, glucocorticoid supplementation, and supportive care. Intravenous fluid therapy was administered using lactated Ringer’s solution (Lactated Ringer’s solution, B. Braun, Melsungen, Germany) at a controlled rehydration rate of approximately 3 mL/kg/h, adjusted according to hydration status, perfusion parameters, and serial electrolyte monitoring. Multimodal adjunctive therapy included maropitant (0.1 mg/kg SC q24h) (Cerenia^®^, 10 mg/mL, Zoetis, Parsippany, NJ, USA) for antiemetic control, gastroprotective treatment with pantoprazole (0.25 mg/kg IV q12h) (Pantoprazo^®^, 40 mg/mL, Rompharm, Bucharest, Romania), parenteral supplementation with vitamins and amino acids using Duphalyte^®^ (30 mL/kg/day IV) (Duphalyte^®^, Zoetis, USA), phosphate-binding therapy with Renal Vet^®^ (1 capsule PO q24h) (VetExpert, Łomianki, Poland), and probiotic support with Purina Pro Plan^®^ FortiFlora^®^ Dog (1 sachet PO q24h) (Nestlé Purina PetCare, St. Louis, MO, USA).

Glucocorticoid therapy was initiated following stabilization using dexamethasone sodium phosphate (0.25 mg/kg IV q24h) (Dexametazone^®^, 4 mg/mL, Rompharm, Bucharest, Romania). Under this therapeutic protocol, serum sodium concentrations increased (127.8 ± 12.25 mmol/L), potassium concentrations decreased (5.57 ± 0.20 mmol/L), and the Na^+^/K^+^ ratio improved (22.99 ± 2.92), consistent with effective restoration of mineralocorticoid activity and improved circulatory volume.

Concurrent reductions in blood urea nitrogen (90.67 ± 111.96 mg/dL), creatinine (2.35 ± 2.85 mg/dL), and phosphorus (8.06 ± 4.00 mg/dL) indicated progressive improvement of renal perfusion and partial resolution of prerenal azotemia. Hemoglobin and hematocrit values decreased during this phase (13.73 ± 4.91 g/dL and 40.75 ± 11.79%, respectively), consistent with hemodilution secondary to controlled volume expansion.

Following clinical stabilization, treatment was continued with oral prednisolone (0.5 mg/kg q24h) (Prednicortone^®^, 20 mg tablets, Dechra, Skipton, UK) in combination with desoxycorticosterone pivalate (DOCP; 2.2 mg/kg SC) (Zycortal^®^, 25 mg/mL, Dechra, Skipton, UK), resulting in sustained improvement of the patients’ general condition.

After discharge (post-discharge phase), all dogs maintained stable biochemical and hematological parameters within or near reference intervals under maintenance replacement therapy. Serum sodium normalized (145.0 ± 7.72 mmol/L), potassium stabilized (4.79 ± 0.14 mmol/L), and the Na^+^/K^+^ ratio increased (30.18 ± 1.53), confirming full restoration of electrolyte balance. Renal parameters returned to physiological ranges (BUN 36.27 ± 13.43 mg/dL; creatinine 0.88 ± 0.31 mg/dL; phosphorus 4.79 ± 0.94 mg/dL), while hemoglobin (15.27 ± 3.29 g/dL) and hematocrit (43.71 ± 9.92%) normalized, reflecting recovery of circulatory homeostasis (Table 2).

### 3.2. Statistical Analysis

Repeated-measure ANOVA (Table 2) demonstrated a significant main effect of treatment phase on the Na^+^/K^+^ ratio (F(2, 4) = 20.46, *p* = 0.0079, *η*^2^*p* = 0.91), confirming the substantial therapeutic impact on electrolyte correction. Sodium and potassium exhibited near-significant improvements (F ≈ 6.8, *p* ≈ 0.05), while BUN, creatinine, and phosphorus showed non-significant but clinically meaningful declines (*p* > 0.05). Hematological indices revealed significant phase-dependent variation for hemoglobin (F(2, 4) = 12.91, *p* = 0.018, *η^2^p* = 0.87) and hematocrit (F(2, 4) = 46.79, *p* = 0.0017, *η*^2^*p* = 0.96), reflecting physiological adjustments to hydration status and corticosteroid-stimulated erythropoiesis.

The F statistic represents the ratio of between-phase variance to within-phase variance, indicating the relative magnitude of treatment effects. The *p* value denotes the likelihood that observed differences occurred by chance, with *p* < 0.05 indicating significance. The partial eta-squared (*η*^2^*p*) quantifies effect size, with higher values denoting stronger therapeutic influence and clinical relevance.

### 3.3. Ultrasonographic Evaluation

Abdominal ultrasonography provided complementary diagnostic insight across all three cases, identifying reactive and reversible parenchymal and structural changes consistent with secondary effects of adrenocortical insufficiency and prerenal azotemia.

In Case 1, ultrasound revealed bilateral renal pyelectasia, reactive abdominal lymphadenopathy, and a mildly thickened gallbladder wall with preserved hepatic and splenic echotexture—findings consistent with functional renal involvement rather than intrinsic pathology.

**Figure 2:**
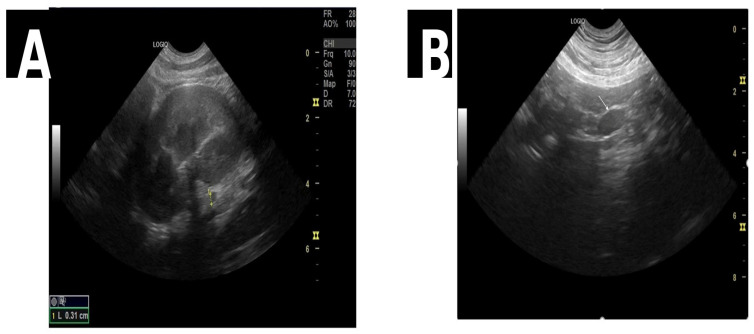
(**A**) Longitudinal view of the left kidney and the caudal lobe of the left adrenal gland, with a reduced short-axis diameter (approximately 0.31 cm). (**B**) Longitudinal view of a reactive mesenteric lymph node (white arrow).

**Figure 3:**
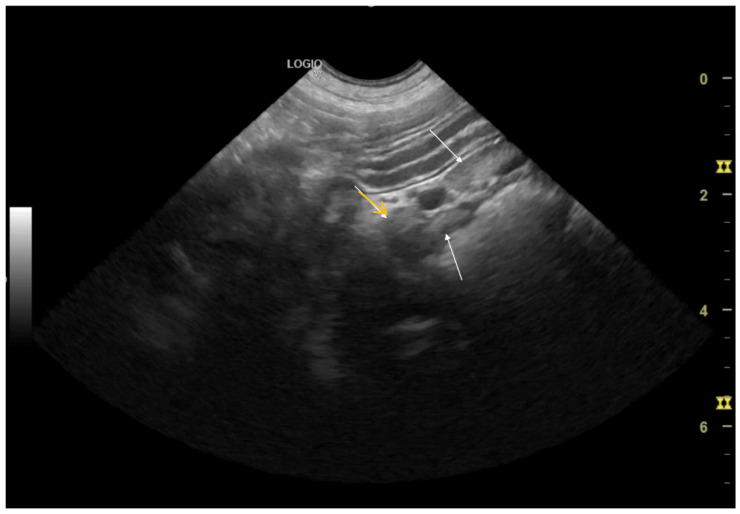
Longitudinal view of the jejunal loop with enlarged adjacent lymph nodes (yellow arrow). Reactive mesenteric lymph nodes (white arrow).

In Case 2, mild bilateral nephromegaly with normal cortical echogenicity (Figure 2A) and reactive mesenteric lymph nodes (Figure 2B and Figure 3) were observed, accompanied by cystitis (bladder wall thickening), without obstructive or degenerative lesions.

**Figure 4:**
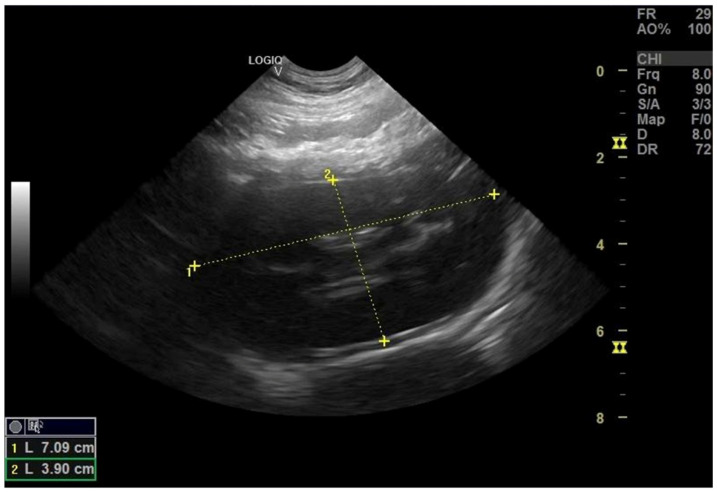
Longitudinal view of the left kidney: pyelectasia, decreased cortical echogenicity.

In Case 3, ultrasound demonstrated bilateral pyelectasia (Figure 4), borderline splenomegaly, and mildly decreased cortical echogenicity, consistent with transient prerenal congestion and preserved renal architecture. Collectively, these findings confirmed the reversibility of renal impairment and the absence of primary renal disease, corroborating biochemical and clinical recovery.

### 3.4. Comparative Analysis: Addisonian Crisis-Associated AKI vs. Non-Endocrine AKI (Retrospective Cohort)

From the total population of dogs evaluated for suspected acute kidney injury (AKI) (n = 34), three dogs were endocrinologically confirmed with primary hypoadrenocorticism and classified as Addisonian crisis-associated AKI. The remaining dogs with AKI and alternative etiologies were classified as non-endocrine AKI (n = 31). Baseline (admission) biochemical parameters were compared between groups to quantify overlap and identify parameters with the greatest discriminatory value (Table 3).

At admission, dogs with Addisonian crisis showed marked hyponatremia and hyperkalemia, resulting in a significantly reduced Na/K ratio compared with dogs with non-endocrine AKI (Welch’s *t*-test, Table 3). All Addisonian dogs (3/3) exhibited a Na/K ratio < 27. In the non-endocrine AKI group, a similarly low Na/K ratio was identified in a small subset of dogs (3/31; 9.7%); however, in these cases, the electrolyte disbalance were secondary to severe concurrent non-endocrine conditions, including post-renal uroabdomen, hypovolemic–ischemic shock, and hepatic dysfunctions. Accordingly, while a Na/K ratio < 27 represented a sensitive screening indicator for Addisonian crisis in this cohort, it was not disease-specific. Fisher’s exact test confirmed a significant association between Na/K < 27 and Addisonian crisis (*p* = 0.0033).

Renal biomarkers (urea/BUN, creatinine) and phosphorus concentrations were higher in dogs with non-endocrine AKI; however, between-group differences did not reach statistical significance, reflecting substantial dispersion and biochemical overlap typical of heterogeneous AKI presentations. Given the very small number of Addisonian cases (n = 3), effect sizes (Hedges’ g) were reported alongside *p*-values to support cautious interpretation without overstatement.

Hematological differences between groups primarily reflected the underlying etiology of AKI. Dogs classified as non-endocrine AKI frequently exhibited anemia and/or leukopenia at presentation, consistent with concurrent infectious, inflammatory, or toxic conditions. In contrast, dogs with Addisonian crisis more commonly showed hemoconcentration, reflected by increased hematocrit and hemoglobin values, likely secondary to hypovolemia. Although these hematological patterns were clinically informative, they did not provide disease-specific discriminatory value for differentiating Addisonian crisis-associated AKI from non-endocrine AKI and were therefore not included as primary comparative variables.

## 4. Complementary Diagnostic Findings

In Case 1, the urinalysis profile was normal, showing no leukocytes, glucose, ketones, or hematuria, trace proteinuria (<15 mg/dL), and specific gravity of 1.030, consistent with preserved tubular concentrating capacity. The urinary pH of 5.0 indicated intact acidification, while microalbumin < 2.5 mg/dL and protein/creatinine ratio 0.2–0.5 confirmed absence of glomerular protein loss. Correlation with normal sediment and transparent urine supported a diagnosis of functional prerenal azotemia secondary to hypovolemia.

Cardiac evaluation revealed sinus rhythm (120 bpm), normal mean electrical axis, and wandering pacemaker pattern without arrhythmias, indicating balanced autonomic tone. Blood pressure remained physiological throughout hospitalization (110/60–150/75 mmHg).

In Case 2, urinalysis parameters remained within normal limits, confirming preserved renal function under therapeutic management. Blood pressure monitoring revealed mild transient hypotension (121/65 mmHg) that normalized within three days (134/60 mmHg). Mild tremors observed at admission prompted a neurological assessment. The patient displayed normal consciousness, coordinated gait, intact proprioception, and symmetric cranial nerve responses. Spinal reflexes (patellar, tibial, sciatic, tricipital, bicipital, and perianal) were normal and bilateral. A localized cervical pain response of probable paravertebral muscular origin was noted, without neurological deficits—consistent with transient metabolic tremor secondary to Addisonian crisis.

In Case 3, urinalysis showed mild proteinuria (+1; 30 mg/dL), elevated urinary creatinine (200 mg/dL), microalbuminuria ≥ 2.5 mg/dL, and specific gravity 1.030, suggesting transient tubular dysfunction due to hypoperfusion during acute adrenocortical insufficiency. The urine was transparent and acellular, excluding infection or hematuria. Electrocardiography revealed sinus rhythm (125 bpm), wandering pacemaker, and normal mean electrical axis without arrhythmias, consistent with parasympathetic predominance. Mild hypotension (111/84 mmHg) was observed at presentation and normalized after isotonic fluid and corticosteroid therapy, confirming cardiovascular stabilization.

Collectively, findings from urinalysis, electrocardiography, blood pressure, and neurological evaluations confirmed the functional and reversible nature of systemic alterations induced by hypoadrenocorticism, excluding primary renal, cardiac, or neurological pathology.

## 5. Discussion

The three canine cases exhibited consistent clinical and biochemical features characteristic of primary hypoadrenocorticism complicated by prerenal acute kidney injury. Clinical presentation—lethargy, anorexia, vomiting, and diarrhea—was typical of Addisonian crisis with secondary hypovolemia (1–6).

Therapeutic management employed a multimodal, pathophysiologic targeted protocol, including isotonic fluid therapy for volume restoration, glucocorticoid (dexamethasone) and mineralocorticoid (desoxycorticosterone pivalate, Zycortal^®^) supplementation, and supportive therapy (antiemetics, gastroprotectives, hepatoprotectives, vitamins, amino acids, phosphate binders, and probiotics). This regimen effectively re-established hemodynamic stability, normalized electrolytes and renal markers, and prevented Addisonian complications. Progressive normalization of biochemical indices—Na (127.8 → 145.0 mmol/L), K (6.72 → 4.79 mmol/L), Na^+^/K^+^ ratio (19.68 → 30.18)—and sustained post-discharge stability confirmed restoration of homeostasis and long-term therapeutic efficacy.

Hypoadrenocorticism remains a major diagnostic challenge in veterinary medicine due to its protean clinical presentation, often termed “the great pretender” [1,18]. In all cases, the combination of hyponatremia, hyperkalemia, low Na^+^/K^+^ ratio, and azotemia paralleled the biochemical pattern of acute renal dysfunction, potentially leading to diagnostic confusion if endocrine testing is omitted [5,25]. The rapid normalization of azotemia following fluid resuscitation and hormone replacement supports a prerenal mechanism driven by hypovolemia and reduced renal perfusion rather than intrinsic renal injury. This finding is consistent with previous reports describing reversible renal dysfunction in Addisonian crisis when promptly treated [21,26]. Restoration of mineralocorticoid activity resulted in normalization of sodium retention and potassium excretion, a hallmark of effective management in primary hypoadrenocorticism [18]. Although decreases in BUN, creatinine, and phosphorus did not reach statistical significance, their consistent downward trend reflects the heterogeneous nature of AKI and the substantial biological variability commonly reported in renal biomarkers [12]. Hematological changes observed during hospitalization were primarily attributable to shifts in hydration status and endocrine correction rather than primary bone marrow pathology. Initial hemoconcentration, reflected by increased hematocrit and hemoglobin values, was consistent with hypovolemia at presentation. Subsequent reductions during hospitalization were compatible with hemodilution following volume expansion, while post-discharge normalization suggested recovery of circulatory homeostasis and erythropoietic balance under corticosteroid replacement therapy. Similar interpretations have been emphasized in the veterinary literature, where hematological parameters are considered supportive but non-specific indicators in endocrine-related renal syndromes [12].

The comparative cohort analysis further demonstrated that while a Na^+^/K^+^ ratio < 27 was highly sensitive for Addisonian crisis in this study (3/3 cases), it lacked disease specificity, as a small subset of dogs with non-endocrine AKI (9.7%) also exhibited similarly reduced ratios, secondary to severe concurrent conditions such as post-renal uroabdomen, hypovolemic–ischemic shock, and advanced hepatic dysfunction. These findings reinforce previous observations that electrolyte abnormalities alone cannot reliably distinguish endocrine from non-endocrine causes of AKI and must be interpreted within the broader clinical and diagnostic context [17].

From a pathophysiological perspective, differentiation between functional prerenal azotemia and intrinsic renal disease remains essential in dogs presenting with azotemia and electrolyte disturbances. Biochemical abnormalities alone may not accurately reflect the presence or absence of structural renal lesions, particularly in the early stages of renal dysfunction [23,24]. Moreover, accurate classification of renal disease in small animals requires an integrated interpretation of clinicopathological data, diagnostic imaging, and dynamic response to therapy [23]. In this study, the absence of persistent ultrasonographic abnormalities and the rapid normalization of renal biomarkers following fluid therapy and hormone replacement strongly support a diagnosis of reversible functional prerenal azotemia rather than primary nephropathy. Collectively, these findings reinforce the need for a multimodal diagnostic approach to avoid misclassification of renal syndromes and to guide appropriate therapeutic decision-making [23,24].

Ultimately, the ACTH stimulation test remained the definitive diagnostic criterion in all cases, confirming persistent cortisol deficiency before and after stimulation [4,6,22]. This study’s findings underscore the necessity of maintaining hypoadrenocorticism as a differential diagnosis in dogs presenting with azotemia, hyponatremia, and hyperkalemia, even when renal parameters appear severely altered. Early endocrine confirmation and prompt initiation of hormone replacement therapy are essential to prevent progression from reversible prerenal dysfunction to irreversible renal injury and to optimize clinical outcomes.

## 6. Conclusions

This retrospective observational study identifies primary hypoadrenocorticism as an important, though infrequent, cause of acute kidney injury in dogs. Addisonian crisis was associated with reversible prerenal azotemia, resulting from hypovolemia and electrolyte imbalance rather than intrinsic renal disease. Electrolyte abnormalities, particularly a reduced sodium-to-potassium ratio, provided the most consistent diagnostic signal at admission; however, this parameter lacked disease specificity. Early endocrine testing and prompt initiation of targeted fluid and hormone replacement therapy led to rapid normalization of biochemical parameters and sustained clinical stability, underscoring the importance of a multimodal diagnostic approach in dogs presenting with suspected AKI.

## Figures and Tables

**Table 1:** Summary of diagnostic methods and paraclinical investigations applied.

Category of Investigation	Method/Parameter Evaluated
General Clinical Examination	Assessment of general condition, vital signs, visible mucous membranes, and hydration status
Hematological Analysis	Complete blood count: erythrocytes, leukocytes, platelets, hematocrit, hemoglobin
Serum Biochemistry	Urea, creatinine, Na^+^, K^+^, Cl^−^, Ca^2+^ (total and ionized), phosphorus, albumin, glucose, hepatic enzymes
Urinalysis	Urine biochemistry, urinary sediment examination
Abdominal Imaging	Renal and adrenal ultrasonography: size, echogenicity, symmetry, corticomedullary ratio
Hormonal Test	ACTH stimulation test (5 µg/kg IV); measurement of serum cortisol concentrations pre- and post-stimulation
Additional Investigations	Rapid tests for vector-borne diseases (e.g., babesiosis); cardiologic examination

**Table 2:** Longitudinal evolution of biochemical and hematological parameters across treatment phases in dogs with Addisonian crisis.

Parameter	Pre-Hospitalization (n = 3)	During Hospitalization (n = 3)	Post-Hospitalization (n = 3)	F	*p*-Value	*η* ^2^ *p*	Interpretation
Na (mmol/L)	127.80 ± 3.12	127.80 ± 12.25	145.00 ± 7.72	6.82	0.0514	0.77	Not significant
K (mmol/L)	6.72 ± 0.99	5.57 ± 0.20	4.79 ± 0.14	6.86	0.0510	0.77	Not significant
Na/K ratio	19.68 ± 1.74	22.99 ± 2.92	30.18 ± 1.53	20.46	0.0079	0.91	Significant
BUN (mg/dL)	130.37 ± 109.82	90.67 ± 111.96	36.27 ± 13.43	0.61	0.5866	0.23	Not significant
Creatinine (mg/dL)	3.05 ± 2.25	2.35 ± 2.85	0.88 ± 0.31	0.70	0.5496	0.26	Not significant
Phosphorus (mg/dL)	9.67 ± 3.11	8.06 ± 4.00	4.79 ± 0.94	1.65	0.3004	0.45	Not significant
Total bilirubin (mg/dL)	0.28 ± 0.04	0.21 ± 0.09	0.15 ± 0.01	5.82	0.1467	0.85	Not significant
Hemoglobin (g/dL)	18.60 ± 3.67	13.73 ± 4.91	15.27 ± 3.29	12.91	0.0180	0.87	Significant
Hematocrit (%)	54.04 ± 12.24	40.75 ± 11.79	43.71 ± 9.92	46.79	0.0017	0.96	Significant

Data are expressed as mean ± standard deviation. Each phase includes three dogs (n = 3). Statistical analysis was performed using repeated-measures ANOVA across the three clinical phases. Partial eta-squared (*η*^2^*p*) indicates effect size. Statistical significance was set at *p* < 0.05.

**Table 3:** Baseline admission biochemistry and electrolyte patterns: Addisonian crisis-associated AKI vs. non-endocrine AKI.

Parameter	Addisonian Crisis–AKI (n = 3) Mean ± SD/n (%)	Non-Endocrine AKI (n = 31 *) Mean ± SD/n (%)	*p*-Value	Effect Size
Na (mmol/L)	127.80 ± 3.12	134.85 ± 9.07	0.026 †	−0.78
K (mmol/L)	6.72 ± 0.99	4.46 ± 0.87	0.049 †	2.52
Na/K ratio	19.68 ± 1.74	31.32 ± 6.26	<0.001 †	−1.87
Na/K < 27, n (%)	3/3 (100%)	3/31 (9.7%)	0.0033 ‡	—
Creatinine (mg/dL)	3.05 ± 2.25	5.93 ± 5.81	0.141 †	−0.50
Urea/BUN (mg/dL)	130.37 ± 109.82	197.97 ± 150.29	0.404 †	−0.45
Phosphorus (mg/dL)	9.67 ± 3.11	14.53 ± 9.74	0.095 †	−0.50

† Welch’s *t*-test. ‡ Fisher’s exact test. * Phosphorus available for 29 dogs in the non-endocrine AKI group.

## Data Availability

The original contributions presented in this study are included in the article. Further inquiries can be directed to the corresponding author.
